# Impact of SARS-CoV-2 vaccination in healthcare workers in a network of clinics in Latin America

**DOI:** 10.1016/j.ijregi.2022.05.002

**Published:** 2022-05-13

**Authors:** David Zarabanda, Sandra Carolina Gonzales, Tsuguhisa Nakayama, Andrea Pascal Moya, Mario Fernando Garzón, Paola Andrea Rengifo, Carlos A. Alvarez-Moreno

**Affiliations:** a Infectious Diseases Department, Clinica Universitaria Colombia, Clinica Colsanitas, Bogotá, Colombia; b Department of Nursing, Clinica Colsanitas, Bogotá, Colombia; c Department of Otolaryngology, Jikei University School of Medicine, Tokyo, Japan; d Department of Human Resources, Clinica Colsanitas, Bogotá, Colombia; e Department of Occupational Health, Clinica Colsanitas, Bogotá, Colombia; f Clinica Colsanitas, Bogotá, Colombia

**Keywords:** COVID-19, SARS-CoV-2, vaccination, Colombia

## Abstract

**Objectives:**

Healthcare workers (HCWs) have been severely affected in terms of both morbidity and mortality since the beginning of the COVID-19 pandemic. During the first few months of 2021, Colombia experienced a surge in positive cases. This study aimed to evaluate the effect of vaccination on the incidence of new positive cases in HCWs.

**Design:**

This was a retrospective cohort study of frontline employees in a network of clinics in Colombia, who were prioritized for COVID-19 vaccination from February to March 2021.

**Results:**

Our findings were consistent with recent reports. During early 2020, the incidence of HCWs positively diagnosed with COVID-19 in Colombia was higher than that for the general population. With the start of the national vaccination program, the incidence among HCWs decreased from April 2021, while that for the general population remained relatively unchanged. Our study identified lower infection rates among HCWs during April (odds ratio [OR], 0.72 [95% CI 0.58–0.90]; *p* < 0.01) and May (odds ratio [OR], 0.25 [95% CI 0.18–0.36]; *p* < 0.01).

**Conclusions:**

COVID-19 vaccination rollout in Colombia during early 2021 led to a decrease in the incidence of new positive cases among HCWs, in contrast to a continuing surge in the general population. Our findings suggested that COVID-19 vaccination provided adequate immunity, which guaranteed protection to HCWs.

## Highlights

• Frontline employees have been severely affected by COVID-19.
• This study assessed the effect of vaccination among healthcare staff in Colombia.
• COVID-19 infections in Colombia increased dramatically during early 2021.
• Vaccination rollout in frontline personnel reduced the incidence of new cases among healthcare workers, in contrast to the general population.

## Introduction

Around 8% of worldwide COVID-19 cases reported up to January 31, 2021 were among healthcare workers (HCWs) (World Health Organization, 2021). The development of messenger RNA (mRNA) vaccines against severe acute respiratory syndrome coronavirus 2 (SARS-CoV-2) is seen as a step forward in containing this pandemic and protecting HCWs (Keehner et al., 2021). However, to the best of our knowledge, minimum data are available to allow evaluation of the impact of vaccination programs in HCWs, subjected to either one or two doses of the vaccine, in a Latin American country. Nevertheless, this region was severely affected by COVID-19 during early 2021, with the predominant variant B.1.621 (Núñez-Zapata et al., 2021).

Using self-reporting data and electronic health records of HCWs, our study aimed to evaluate the effects of vaccination and the incidence of SARS-CoV-2 infection among vaccinated staff in a network of clinics in Colombia.

## Methods

This retrospective cohort study was conducted across the Clinica Colsanitas network in Colombia, which has a total of 12 190 employees. HCWs were prioritized for vaccination with BNT162b2 (Pfizer-BioNTech COVID-19 mRNA), according to Colombia's national vaccination program (Ministerio de Salud de Colombia, 2021).

To identify the vaccinated HCWs and new COVID-19 cases, occupational health records were reviewed. As per the institutional policy, for all HCWs presenting with typical symptoms suggestive of COVID-19, such as fever, cough, and change/loss of taste or smell, along with other symptoms, including shortness of breath, sore throat, runny nose, headache, muscle aches, extreme fatigue, or diarrhea, or reporting potential SARS-CoV-2 exposure, an RT-PCR assay using nasopharyngeal swabs was performed to confirm the diagnosis. Of note, prevention and control strategies, mask wearing, physical distancing, and other non-pharmaceutical measures had been implemented before vaccination rollout and did not vary during the study period.

Vaccination status was determined at the time of first SARS-CoV-2 positive test on or after February 18. Non-vaccinated employees were those with no record of vaccine on file. Partially vaccinated employees were those who had received one dose or the second dose of the BNT162b2 vaccine less than 7 days before the date of diagnosis. Fully vaccinated employees were those who had received a second dose of the BNT162b2 vaccine at least 7 days before the date of diagnosis.

No institutional review board approval was required because all the study subjects were de-identified. The data were also used to analyze the infection incidence in frontline employees from April 6, 2020 to May 19, 2021. Data on the number of positive cases in the general population were extracted from OurWorldinData.org (Ritchie et al., 2020). Chi-squared tests were performed, with *p* < 0.05 considered to be statistically significant. Data analysis was performed using GraphPad Prism version 6.0 (GraphPad Software, San Diego, CA).

## Results

**Fig. 1:**
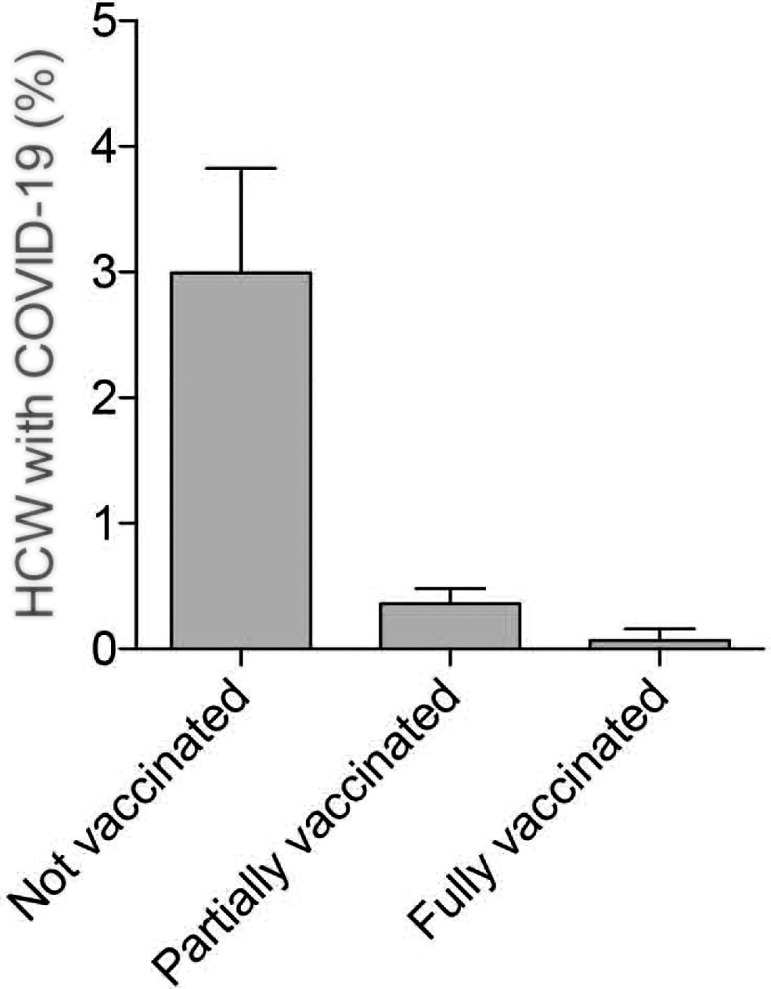
The percentage of new SARS-CoV-2 infections among 12 190 healthcare workers (HCWs) prioritized for vaccination in phase one of the vaccination drive across the Clinica Colsanitas network in Colombia. All data have been stratified according to vaccination status from February 18, 2021 to March 31, 2021. (*p*-value < 0.01 by chi-squared test)

During the first month of the vaccination program, which started on February 18, 2021, 8924 (73.2%) HCWs received the first dose of the vaccine, and 2978 (24.4%) received both doses. Up to March 31, 2021, a total of 82 employees (0.67%) eligible to receive a vaccine had been diagnosed with COVID-19 infection. Of those HCWs who had not received any dose, 2.99% (48/1604) were infected; of those who were partially vaccinated, 0.36% (32/8924) were infected; and among those who were fully vaccinated, only 0.07% (2/2978) were infected (*p* < 0.01) (Fig. 1). The relative risk (RR) was increased for non-vaccinated HCWs (RR 5.05 [95% CI 4.18–6.10]; *p* < 0.01).

**Fig. 2:**
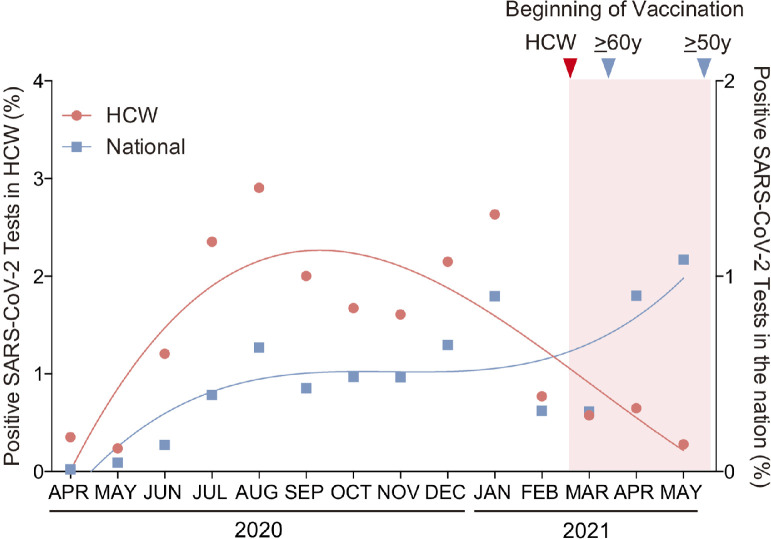
SARS-CoV-2 positive test rates in the general populations vs healthcare workers (HCWs), showing a higher positive rate in HCWs (red circles) than in the general population (blue squares) before the vaccination started for HCWs (February 18, 2021). After the start of vaccinations for HCWs, the incidence of positive cases decreased to a lower level than in the general population. The solid lines show the best-fit curves (third order polynomial). In the general population, vaccination commenced for people 60 years of age or older on March 8, 2021, and for those 50 years of age and older on May 22, 2021.

The infection trend for COVID-19 was further investigated from April 6, 2020, to May 19, 2021. (Up to March 2021, the incidence of positive cases among HCWs had been higher than that of Colombia's general population (*p* < 0.01).) However, despite a rapid increase in positive cases in the general population from April 2021, our study identified lower infection rates among HCWs over the same period (odds ratio [OR] 0.72 [95% CI 0.58–0.90]; *p* < 0.01) and from May 2021 (odds ratio [OR] 0.25 [95% CI 0.18–0.36]; *p* < 0.01) (Fig. 2).

## Discussion

The overall outcome of our study suggested a lower incidence of SARS-CoV-2 infection in more exposed frontline HCWs after the vaccine rollout on February 18, 2021. The findings of our study were consistent with those of previous reports (Benenson et al., 2021; Gupta et al., 2021; Keehner et al., 2021). Vaccine effectiveness has been assessed in other studies and regions; for example, Israel and the UK have also studied the effects of one or two doses of the vaccine, but limited information is available for South America (Haas et al., 2021; Hall et al., 2021; Vasileiou et al., 2021). In Colombia specifically, before the start of vaccination, the rate of positive diagnoses among HCWs was higher than for the general population, but there was a clear reversal of this situation after the vaccination rollout.

This study had some limitations. First, it was not possible to assess the rates of prior infection in frontline personnel before the vaccination rollout and thus adjust the data for potential confounders. Second, testing protocols, accessibility, and frequency for the general population were most likely different and limited when compared with those for HCWs. Furthermore, the self-reported assessment implemented to gather data from HCWs could still have influenced our estimates. Finally, the retrospective observational study design would have limited the possibility of extrapolating the findings to other settings with different demographic characteristics.

In addition to the vaccine rollout, implementation of prevention and protection measures in HCWs can also be helpful in managing COVID-19 infections. According to the literature, these strategies often include: the implementation of a reporting system to monitor all HCWs; training and careful compliance with correct personal protective equipment (PPE) utilization, with a high- or low-risk assessment after hazardous contacts for early identification and isolation of symptomatic workers; and strict social distancing and avoidance of face-to-face contacts within colleagues (Liu et al., 2020; Vimercati et al., 2021).

Considering the surge of COVID-19-positive cases in Latin America, especially in Colombia, during early 2021, these results further emphasize that COVID-19 vaccination provides an adequate level of immunity, which guarantees protection for HCWs. Furthermore, this strategy has allowed essential and frontline HCWs to reduce their risk of infection and transmission to patients, with fewer requirements for quarantine, testing, and tracing within critical-care services.
